# ﻿Drought stress responses revealed by genomic and transcriptomic analyses of two macrofungi (*Inonotus hispidus* and *Inocutis levis*) from *Populus euphratica*

**DOI:** 10.3897/imafungus.16.163859

**Published:** 2025-09-15

**Authors:** Miao Zhou, Meng-Xue Lv, Dong-Mei Wu, Neng Gao, Tai-Min Xu, Yi-Fei Sun, Bao-Kai Cui

**Affiliations:** 1 State Key Laboratory of Efficient Production of Forest Resources, School of Ecology and Nature Conservation, Beijing Forestry University, Beijing 100083, China Beijing Forestry University Beijing China; 2 Biotechnology Research Institute, Xinjiang Academy of Agricultural and Reclamation Sciences / Xinjiang Production & Construction Group Key Laboratory of Crop Germplasm Enhancement and Gene Resources Utilization, Shihezi, Xinjiang 832000, China Biotechnology Research Institute, Xinjiang Academy of Agricultural and Reclamation Sciences / Xinjiang Production & Construction Group Key Laboratory of Crop Germplasm Enhancement and Gene Resources Utilization Shihezi China

**Keywords:** Drought-resistant, gene expression, *Inonotus hispidus*, *Inocutis levis*, whole genome sequencing

## Abstract

*Populus euphratica* is a key deciduous and tall arbour capable of forming forests in arid and desert environments, exhibiting notable tolerance to drought, salinity and bacterial resistance. This study completed whole-genome sequencing of *Inonotus hispidus* and *Inocutis levis*, collected from Xinjiang, China, to predict genome structure and identify potential drought-related genes. Combined with transcriptome sequencing under different drought conditions simulated using PEG-6000, the gene expression regulation during drought tolerance was analysed. Whole-genome sequencing was carried out on the Illumina Novaseq and Pacbio Sequel platforms, resulting in genome size of 34.57 Mb for *Inonotus hispidus* and 37.17 Mb for *Inocutis levis*, respectively. A total of 10,169 and 10,140 protein-coding genes were annotated in these two species. The genomes of two species exhibited high synteny with 7,226 shared homologous genes and their functional annotations showed high similarity. Under drought stress at three PEG-6000 concentrations (10%, 30% and 50%), the transcriptomic analyses revealed 4,550 and 2,113 differentially expressed genes (DEGs) in the two fungi, respectively, with an increasing number of up- and down-regulated genes as the drought stress intensified. Gene expression profiles in response to drought stress showed prominent changes, amongst which the genes related to antioxidation, osmotic regulation, signal transduction and ribosomal function may play important roles. In the ribosome pathway, *Inonotus hispidus* showed a significant down-regulation of ribosomal-related genes under mild drought stress, which is up-regulated once again as the stress intensifies, while *Inocutis levis* exhibited significant up-regulation of these genes under severe drought stress, highlighting distinct drought adaptation strategies. This study provides essential theoretical insights into the molecular adaptation mechanisms of fungi in dry environments and offers new perspectives for the development of microbial resources in arid regions.

## ﻿Introduction

Drought is one of the most destructive environmental stresses globally, severely limiting the growth and development of plants and fungi, often leading to significant economic losses in agricultural and forestry settings (Anjum et al. 2011; Begum et al. 2019). In drought ecosystems, *Populus euphratica* Oliv. is a characteristic tree species with exceptional drought resistance, capable of surviving and reproducing under harsh environmental conditions like water scarcity and saline soils (Li et al. 2011). Drought conditions inhibit the proliferation of microorganisms such as fungi, bacteria and viruses and only few wood-inhabiting fungi can grow on *P. euphratica* (Qiao et al. 2008).

In this study, two strains of *Inonotus hispidus* (Bull.) P. Karst. and *Inocutis levis* (P. Karst.) Y.C. Dai & Niemelä belonging to the *Hymenochaetaceae* (*Hymenochaetales*, *Basidiomycota*) were collected from Xinjiang, China from wild *P. euphratica* trees. *Inonotus hispidus* and *Inocutis levis*, as white-rot fungi, may cause early tree weakness with finite pathogenicity (Zan et al. 2011; Dai 2012). *Inocutis* Fiasson & Niemelä was separated in 1984 from *Inonotus* P. Karst., which has similar morphological characters (Wu et al. 2022) and homologous medicinal values, such as anti-tumour (Vinogradov and Wasser 2005; Li and Bao 2022), anti-hyperglycaemic (Ehsanifard et al. 2019; Liu et al. 2019b), antioxidant and antimicrobial properties (Wang et al. 2022b; Chaharmiri-Dokhaharani et al. 2023). These strains originated from dry environments and exhibited high survival rates under prolonged water deficient conditions in preliminary screenings, making them ideal model systems for dissecting fungal drought adaptation mechanisms.

Three complete genome sequences of *Inonotus hispidus* have already been reported (Tang et al. 2022; Zhang et al. 2022; Wang et al. 2023). These studies have identified the candidate genes associated with polysaccharide synthesis, carbohydrate-active enzymes and secondary metabolite biosynthesis. The differences in the genetic basis of *Inonotus hispidus* growing on mulberry and poplar, improves our understanding of its biosynthetic pathways, high-yield cultivation and medicinal values. However, no omics studies have been reported for *Inocutis levis* to date and its genetic information remains to be explored.

Drought stress triggers a series of complex physiological and molecular responses in organisms. At the cellular level, drought leads to cellular dehydration, causing a decrease in turgor pressure, which subsequently affects normal metabolism and physiological functions (Seleiman et al. 2021). For instance, the enzyme activity within many plant and fungal cells will be changed due to water shortage; some enzymes are inhibited, while other activities associated with antioxidant defence, such as superoxide dismutase (*SOD*) and catalase (*CAT*), may be induced to eliminate reactive oxygen species (*ROS*) to prevent oxidative damage to cells (Das and Roychoudhury 2014). At the genetic level, drought stress can induce significant changes in gene expression. Genes involved in the synthesis of osmotic regulators, such as proline and trehalose, are up-regulated to synthesise compatible solutes that regulate intracellular osmotic pressure and maintain cellular water balance (Garg et al. 2002; Singh et al. 2015). Additionally, the genes related to the synthesis and modification of cell walls and membranes may also play a role in drought adaptation by altering compositions, to enhance the cell’s ability to retain water and its mechanical strength (Gall et al. 2015; Yu et al. 2021). For fungi, drought also can affect the growth rate of hyphae and fruiting bodies, promoting the formation of more compact structures or the production of specialised dormant structures to reduce water loss and improve survival in drought environments (Ma et al. 2014). *Inonotus hispidus* and *Inocutis levis* may also have similar mechanisms to resist drought stress.

Some progress has been made in studying the drought adaptation for fungi using transcriptome sequencing techniques. The lichen-forming fungus *Endocarpon pusillum* Hedwig exhibited strong drought resistance and the transcriptomic analyses revealed that the damage repair, energy supply and carbon metabolism jointly contributed to its drought adaptation (Wang et al. 2015). Transcriptomic analyses of *Auricularia fibrillifera* Kobayasi under drought stress revealed significant enrichment of pathways including tyrosine metabolism, caffeine metabolism, ribosome, phagosome, proline and arginine metabolism, peroxisome and MAPK signalling. Its strong drought tolerance was attributed to enhanced reactive oxygen species (*ROS*) scavenging, osmotic regulation, signal transduction, cell wall re-modelling and phagocytosis (Wang et al. 2022a). Similarly, in *Cenococcum geophilum* Fries, comparative transcriptomics of six strains under 10% polyethylene glycol (PEG) treatment showed that drought-sensitive strains alleviated drought stress through Na^+^ and K^+^ ion uptake for osmotic adjustment and up-regulation of peroxisome pathway genes to enhance antioxidant enzyme activity (Li et al. 2022). Drought-tolerant strains responded to drought stress by up-regulating functional genes involved in ubiquinone and other terpene quinone biosynthesis and sphingolipid metabolism pathways. Overall, although some studies have been conducted on drought adaptation of fungi, the understanding of drought tolerance mechanisms from a genetic perspective remains limited and broader inclusion of fungal taxa with more comprehensive analyses are required.

To further understand the drought tolerance mechanisms of *Inonotus hispidus* and *Inocutis levis*, this study utilised the whole-genome and transcriptome sequencing to explore the genomic features, compare the gene expression profiles under varying drought stress levels and predict the key genes and metabolic pathways during the drought adaptation progress. The findings of this study will help explain the growth characteristics of these two fungi at the genetic level, assist in their artificial cultivation and provide the foundational data for the exploration and utilisation of fungal resources in arid regions.

## ﻿Materials and methods

### ﻿Fungal strains

The experimental strains of *Inocutis levis* and *Inonotus hispidus* were collected from wild *P. euphratica* trees in Luntai County, Xinjiang, China (41°46'39"N, 84°15'39"E) in 2020 and 2022, respectively and isolated from the fruiting bodies directly. The annual average precipitation is 45.2 mm and annual evaporation rate is 1887–2910 mm in this region (https://gisrs.cn). Through microscopic examination, the dikaryotic strains were purified and are currently preserved in the Institute of Microbiology, Beijing Forestry University (BJFC), noted as *Inonotus hispidus* Wu 2022-1 and *Inocutis levis* Cui 19065. The strains were preliminarily cultured and activated on Potato Dextrose Agar (PDA) medium in the dark at 28 °C for 7 days.

### ﻿Genome sequencing and assembly

For genome sequencing, the strains were inoculated in liquid medium (glucose 20.0 g/l, yeast extract 5.0 g/l, KH_2_PO_4_ 1.0 g/l, MgSO_4_·7H_2_O 0.5 g/l, VB_1_ 0.01 g/l) and cultured in a shaker flask at 28 °C and 150 rpm/min for 7 days. The mycelia were collected by filtration under aseptic conditions and quickly frozen in liquid nitrogen for over 10 minutes, then stored at –80 °C. Genomic DNA was extracted using an improved CTAB method (Fulton 1995). The DNA concentration, quality and integrity were assessed using a Qubit Fluorometer (Invitrogen, USA) and a NanoDrop Spectrophotometer (Thermo Scientific, USA). The samples were sent to Personal Biotechnology Co., Ltd. (Shanghai, China) for library construction with different insert sizes using the Whole Genome Shotgun (WGS) approach. The genomes of the two fungi were sequenced using the Illumina Novaseq platform and the Pacbio Sequel platform with 400 bp and 10 kb insert sizes, respectively.

AdapterRemoval v.2.3.4 (Schubert et al. 2016) and SOAPec v.2.03 (Luo et al. 2012) were used to remove the adapter contamination and perform data correction, then the high-quality sequences were filtered for genome assembly and quality assessment. JELLYFISH v.2.3.0 was employed to calculate the depth distribution map of K-mers (Marçais and Kingsford 2011). Falcon v.0.3.0 (Chin et al. 2016) and CANU v.1.7.1 (Koren et al. 2017) were used to perform *de novo* assembly of PacBio long-read sequences to generate contigs and scaffolds. The resulting assembly was then polished with Illumina short reads using pilon v.1.24 (Walker et al. 2014) to obtain the final genome sequence. BUSCO v.5.4.5 (Benchmarking Universal Single-Copy Orthologs, http://busco.ezlab.org), based on the fungi_odb10 database, was used to evaluate the completeness of the genome assembly (Manni et al. 2021).

### ﻿Genome annotation

Repetitive sequences were identified through *de novo* prediction using RepeatModeler v.2.0.5 (Flynn et al. 2020). The results were merged with the Repbase database to create a comprehensive species-specific repeat library. Subsequently, the RepeatMasker v.4.1.6 software (Tarailo-Graovac and Chen 2009) was utilised (parameters: -e ncbi -s -gff -xsmall) to analyse the repetitive sequences in the genome.

Transfer RNA (tRNA) genes were predicted using tRNAscan-SE v.2.0 in the eukaryotic covariance model (Chan et al. 2016), while ribosomal RNA (rRNA) genes were identified with RNAmmer v.1.2 and integrated with HMMER3 (Lagesen et al. 2007). Other non-coding RNAs (i.e. snRNAs, snoRNAs, miRNAs) were predicted by comparing the genome to the ncRNA annotation database Rfam v.14.1 applying an E-value threshold of < 1e^-5^ (Griffiths-Jones 2004).

Protein-coding gene prediction followed a three-step process: First, gene models were predicted *de novo* using Augustus v.2.5.5, glimmerHMM v.3.0.4 and GeneMark-ES v.4.71 (Majoros et al. 2004; Stanke and Morgenstern 2005; Ter-Hovhannisyan et al. 2008) to generate initial gene predictions. Next, homologous genes were identified by aligning the protein sequences of closely-related species using Exonerate v.2.2.0 (Slater and Birney 2005). Finally, the results of de-novo predictions and homology-based predictions were integrated using EVidenceModeler v.2.0.0 (Haas et al. 2008).

Interspecific and intraspecific synteny analyses within and between *Inonotus hispidus* and *Inocutis levis* was performed to present the duplication and similarity of two species. The TBtools-II software (Chen et al. 2023) was used to create a Circos plot and conduct collinearity analyses for genome visualisation. Orthologous gene analyses between the two fungal species were executed using OrthoVenn3 (Sun et al. 2023) with OrthoMCL parameters, applying an E-value threshold of < 1e^-5^. The Ks values of collinear gene pairs were computed by means of the Simple Ka/Ks Calculator within this software and a Ks density plot was generated in R v.4.4.1 (https://cran.r-project.org/bin/windows/base/old/4.4.1/) to reveal potential whole-genome duplication events.

### ﻿Gene functional annotation

Functional annotation of protein-coding genes in *Inonotus hispidus* and *Inocutis levis* were performed using Diamond v.2.0.14 (Buchfink et al. 2015) and Blast+ v.2.5.0 (Camacho et al. 2009). The annotated genes were aligned against various databases with default parameters applied and the best hit was selected for functional annotation. The databases used in this study were: NR v.4 (Non-Redundant Protein Sequence Database, http://ftp.ncbi.nih.gov/blast/db/), EggNOG v.6.0 (Evolutionary genealogy of genes: Non-supervised Orthologous Groups, http://eggnogdb.embl.de/#/app/home/), KEGG v.105.1 (Kyoto Encyclopedia of Genes and Genomes, http://www.genome.jp/kegg/), Swiss-Prot v.2017_01 (Swiss Protein Database, http://www.uniprot.org/), GO v.2023-01-01 (Gene Ontology, http://www.geneontology.org/), cytochrome P450 v.2013-08-04 (http://p450.riceblast.snu.ac.kr/index.php?a=view), TCDB v.2021 (Transporter Classification Database, http://www.tcdb.org/), Pfam v.35.0 (Protein Family Database, http://pfam.xfam.org/) and PHI v.4.15 (Pathogen Host Interactions Database, http://www.phi-base.org/). The genes related to CAZymes, were predicted using HMMscan v.4.1 through CAZymes v.2023 (Carbohydrate-Active enzymes Database, http://www.cazy.org/). Signal peptide prediction was conducted using SignalP 5.0 (Armenteros et al. 2019), while transmembrane domain predictions were made using TMHMM v.2.0 (Chen et al. 2003).

### ﻿Transcriptome sequencing and analyses

Polyethylene glycol 6000 (PEG-6000) is a non-toxic, colourless and odourless polymer and widely used as an osmotic agent, being employed to simulate drought stress conditions (Wang et al. 2015). For RNA-seq analyses, *Inonotus hispidus* and *Inocutis levis* were grown in PDA medium, supplemented with 0% (CK), 10% (P10), 30% (P30) and 50% (P50) PEG-6000. The different concentrations of PEG-6000 represented no drought and mild, moderate and severe drought, respectively. These groups were then cultivated at 28 °C and 150 rpm/min for 10 days, with each treatment being performed in triplicate for each species.

The 10-day old mycelia of 24 sample sets (12 per species) were harvested and sent to Personal Biotechnology Co., Ltd. (Shanghai, China) for transcriptome sequencing. Total RNA was extracted using the Trizol Reagent (Invitrogen Life Technologies) following manufacturer’s protocol, then the concentration, quality and integrity were determined using a NanoDrop spectrophotometer (Thermo Scientific). Sequencing of total RNA was performed on the Illumina NovaSeq platform at Personal Biotechnology Co., Ltd. Raw reads were quality-filtered using fastp v.0.22.0 (parameters: --qualified_quality_phred 20 --length_required 50 --trim_poly_x) to obtain high quality sequences for further analyses (Chen et al. 2018).

Filtered mRNA reads were mapped to the genome of each species using HISAT2 v.2.1.0 for strand-specific mapping (Kim et al. 2019). HTSeq v.0.9.1 (Anders et al. 2015) was used to compare the Read Count values on each gene as the original expression of the gene and FPKM (Fragments Per Kilobase of exon model per Million mapped fragments) was used to standardise the expression. Based on FPKM expression data, the R package pheatmap v.1.0.13 (Kolde 2025) was used to perform hierarchical clustering analysis on the differentially expressed genes. Count normalisation and differential expression analysis was performed in the R package DEseq2 v.1.48.1 (Wang et al. 2010) identifying differentially expressed genes (DEGs) with adjusted P-value < 0.05 and |log2Foldchange| > 1 as thresholds. Further GO and KEGG pathway enrichment analyses of the DEGs were performed using the package topGO v.2.50.0 (Alexa and Rahnenfuhrer 2006) and the package clusterProfiler v.4.6.0 (Yu et al. 2012), focusing on the significant enrichment pathway with P-value < 0.05.

## ﻿Results

### ﻿Genome assembly and annotation

The sequencing information of *Inonotus hispidus* and *Inocutis levis* are shown in Suppl. material 2: tables S1, S2. The K-mer analyses for *Inonotus hispidus* and *Inocutis levis* revealed two distinct peaks in their respective curves, indicating genome heterozygosity rates of 1.51% for *Inonotus hispidus* and 1.62% for *Inocutis levis* (Suppl. materials 1, 2: fig. S1, table S3).

The genome of *Inonotus hispidus* were assembled into 41 scaffolds, resulting in a genome size of 34.57 Mb (Table 1). For *Inocutis levis*, 115 scaffolds were obtained in a genome size of 37.17 Mb. Both genome assemblies contained 13 scaffolds longer than 1 Mb, the L50 and L90 were greater than 1 Mb and the percentages of complete BUSCOs for each exceeded 96%.

**Table 1.** Genome statistics of *Inonotus hispidus* and *Inocutis levis*.

Parameter	Inonotus hispidus Wu 2022-1	Inocutis levis Cui 19065
Total sequence length (bp)	34,568,596	37,171,511
Total scaffold number	41	115
L50 (bp)	2,927,195	2,730,682
N50 Number	5	5
L90 (bp)	1,461,081	1,437,238
N90 Number	13	13
Min sequence length (bp)	9,037	11,206
Max sequence length (bp)	4,181,198	4,783,972
GC content (%)	48.1031	46.1964
Scaffolds greater than 1kb	41	115
Scaffolds greater than 1Mb	13	13
Complete BUSCOs	97.30%	96.60%
Complete and single-copy BUSCOs	94.50%	96.30%
Complete and duplicated BUSCOs	2.80%	0.30%
Fragmented BUSCOs	0.20%	0.30%

There were 2.10% (725,831 bp) repetitive sequences in *Inonotus hispidus* and 4.76% (1,770,469 bp) in *Inocutis levis* (Suppl. material 2: table S4). Amongst interspersed repeats, LTRs, accounting for 1.28% (443,892 bp) and 3.54% (1,315,335 bp), respectively, were the most enriched in the two genomes and LINEs and DNA transposons all accounted for less than 0.1% (20,088 bp for *Inonotus hispidus* and 33,856 bp for *Inonotus hispidus*). No SINEs were annotated in either genome. Amongst other repeats, simple repeats accounting for 0.56% (191,951 bp) and 0.81% (300,444 bp), were the most enriched and the rolling-circles, small RNA, satellites and low complexity all accounted for 0.20% (69,710 bp) and 0.32% (122,378 bp). The non-coding RNAs accounted for 0.12% (42,846 bp) and 0.46% (169,715 bp) of the total genome in each fungus, including 8,454 bp/75,554 bp ncRNA, 555 bp/999 bp 5S rRNA, 760 bp/1,216 bp 5.8S rRNA, 9,769 bp/28,999 bp 18S rRNA, 9,769 bp/28,999 bp 28S rRNA and 7,959 bp/30,395 bp tRNA (Suppl. material 2: table S5). The prediction of protein-coding genes was integrated from *de novo* and homology-based predictions from closely-related species. A total of 10,169 protein-coding genes were predicted in *Inonotus hispidus*, accounting for 52.22% of its genome. In *Inocutis levis*, 10,140 protein-coding genes were identified, representing 48.39% of its genome (Table 2).

**Table 2.** Protein-coding genes in *Inonotus hispidus* and *Inocutis levis*.

Property	Inonotus hispidus Wu 2022-1	Inocutis levis Cui 19065
Total gene length (bp)	18,051,469	17,985,641
Genes percentage of genome (%)	52.22%	48.39%
Total genes	10,169	10,140
Average gene length (bp)	1,775.1	1,773.7
Total exons	56,681	54,072
Average exons per gene	5.5	5.3
Total exons length (bp)	14,815,196	15,033,424
Exons percentage of genome (%)	42.86%	40.44%
Average exon length (bp)	261.3	278
Average intron length (bp)	69.5	67.1
Total CDS length (bp)	14,815,196	15,033,424
CDS percentage of genome (%)	42.86%	40.44%
Average CDS length (bp)	1,456.8	1,482.5

**Figure 1.**
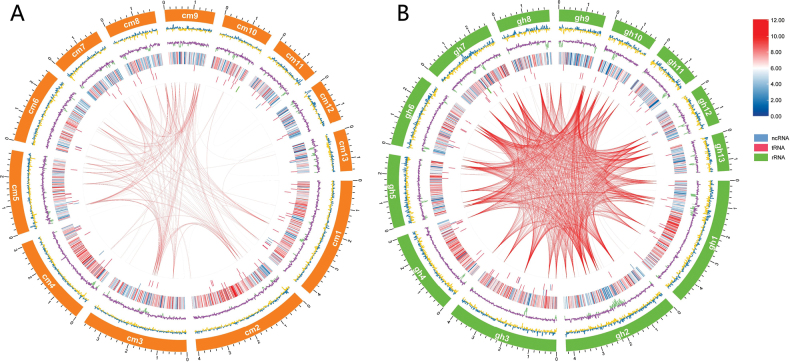
Genome Circos plot of scaffolds longer than 1Mb. A *Inonotus hispidus*; B *Inocutis levis*. From outside to inside: scale, GC skew (the specific algorithm = (G−C)/(G + C); blue: ≥ 1, yellow: < 1), GC content (purple: ≥ 46%, green: < 46%), gene density, ncRNA on positive chain, ncRNA on negative chain, genome duplication (regions with sequence similarity greater than 10 kb are connected by red lines).

Only sequences longer than 1 Mb were presented in Circos plots, which visually illustrated the genomic structural features of the two species (Fig. 1). Moving from the outside in, this circular genome visualisation began with an outer scale ring indicating 13 sequences longer than 1 Mb. The next two rings displayed GC skew and GC content, respectively, both showing significant regional deviations in the two fungal species. The fourth ring illustrated gene density, transitioning from red (high density) to blue (low density) and, notably, the areas of low GC content directly corresponded to these gene-sparse regions. The fifth and sixth rings plotted the locations of non-coding RNAs on the positive and negative strands. Finally, the inner portion featured red lines connecting syntenic gene pairs, highlighting homologous genomic segments larger than 10 kb. *Inocutis levis* showed a greater number of collinear regions within its genome, indicating a higher frequency of gene duplication events.

**Figure 2.**
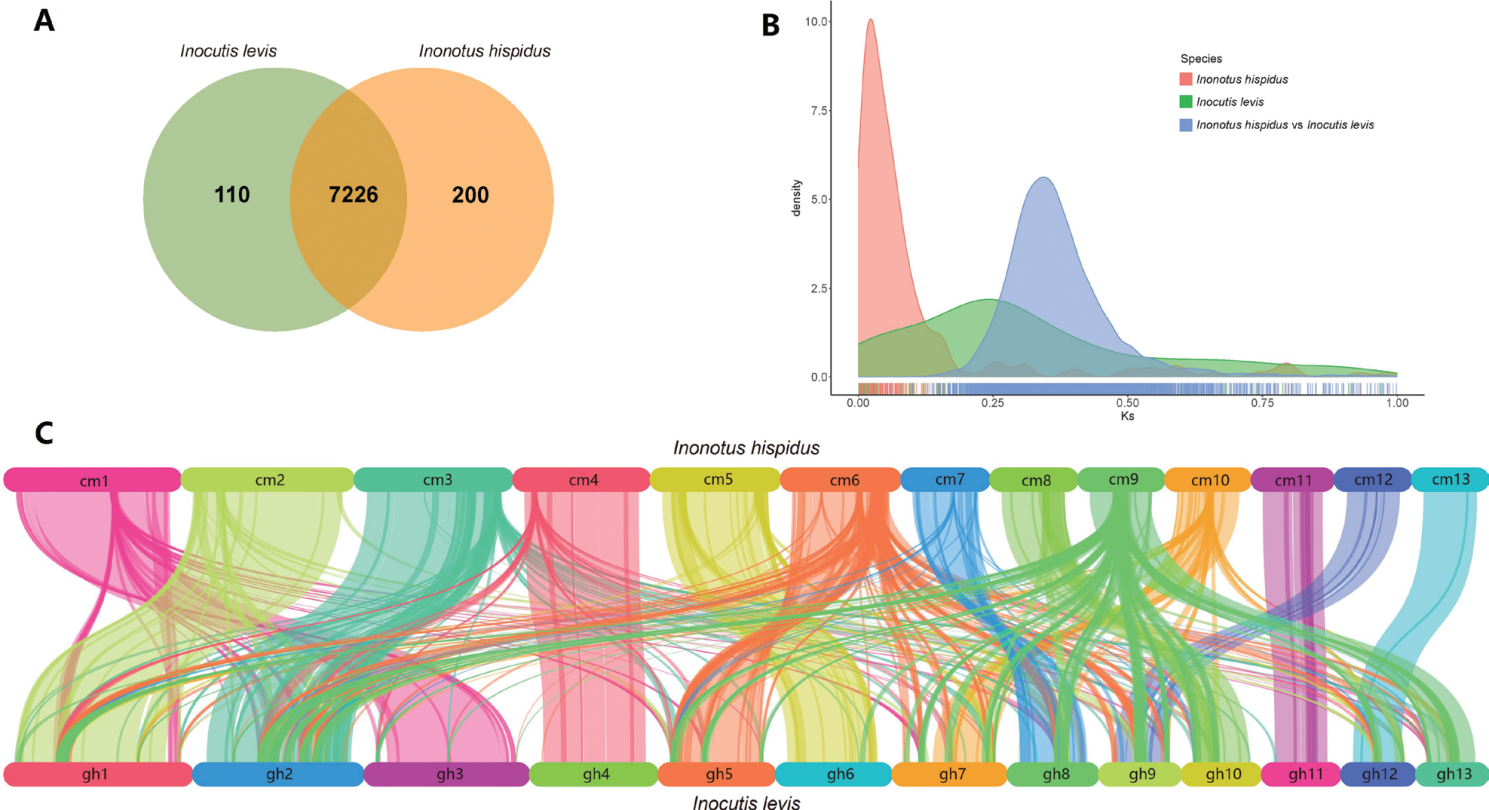
Comparative genomic analyses of *Inonotus hispidus* and *Inocutis levis*. A Orthologous gene analyses presented in Venn plot; B Illustration of Ks density curve; C Interspecific similarity in collinearity analyses, different colours represent 13 scaffolds.

Orthologous gene analyses identified 7,226 shared homologous genes between *Inonotus hispidus* and *Inocutis levis* (Fig. 2A), with the former possessing more unique homologues (200 vs. 110). The Ks curve indicated neutral evolutionary rates, with peak positions revealing genome duplication timing (Fig. 2B). Both species showed a single low Ks peak, indicating conserved gene retention without genome duplication. *Inonotus hispidus* exhibited a sharp peak near Ks = 0, while *Inocutis levis* displayed a broader, flatter peak, suggesting more dispersed synonymous substitutions, potentially from diverse selective pressures or higher historical mutation rates. The interspecies Ks peak at 0.35 (blue region) indicated recent divergence and genetic similarity. Collinearity analysis revealed 14,138 collinear gene pairs and 69.61% syntenic regions, with sequence insertions or re-arrangements reflecting evolutionary divergence (Fig. 2C).

### ﻿Genome functional annotation

All protein-coding genes predicted in *Inonotus hispidus* and *Inocutis levis* were subjected to sequence similarity analysis and motif similarity search, based on ten public databases to obtain a comprehensive functional annotation. *Inonotus hispidus* and *Inocutis levis* had 10,141 and 10,095 genes (99.72% and 99.56%, respectively) that aligned with at least one database (Table 3). Overall, the annotation outcomes of *Inonotus hispidus* and *Inocutis levis* exhibited high similarities.

**Table 3.** Functional annotation, based on ten databases.

Database	Inonotus hispidus Wu 2022-1		Inocutis levis Cui 19065	
	Number	Percentage (%)	Number	Percentage (%)
NR^1^	9,452	92.95	9,380	92.50
EggNOG^2^	7,775	76.46	7,813	77.05
KEGG^3^	3,571	35.12	3,347	33.01
SwissProt^4^	6,099	59.98	6,197	61.11
GO^5^	5,824	57.27	6,024	59.41
P450^6^	9,917	97.52	9,908	97.71
CAZymes^7^	418	4.11	412	4.06
TCDB^8^	1,336	13.14	1,263	12.46
Pfam^9^	6,821	67.08	6,825	67.31
PHI^10^	2,068	20.34	1,898	18.72
Total	10,141	99.72	10,095	99.56

^1^ NR v.4: Non-Redundant Protein Sequence Database, http://ftp.ncbi.nih.gov/blast/db/. ^2^ EggNOG v.6.0: Evolutionary genealogy of genes: Non-supervised Orthologous Groups, http://eggnogdb.embl.de/#/app/home/. ^3^ KEGG v.105.1: Kyoto Encyclopedia of Genes and Genomes, http://www.genome.jp/kegg/. ^4^ Swiss-Prot v.2017_01: Swiss Protein Database, http://www.uniprot.org/. ^5^ GO v.2023-01-01: Gene Ontology, http://www.geneontology.org/. ^6^ P450 v.2013-08-04: cytochromeP450, http://p450.riceblast.snu.ac.kr/index.php?a=view. ^7^ CAZymes v.2023: Carbohydrate-Active enzymes Database, http://www.cazy.org/. ^8^ TCDB v.2021: Transporter Classification Database, http://www.tcdb.org/. ^9^ Pfam v.35.0: Protein Family Database, http://pfam.xfam.org/. ^10^ PHI v.4.15 : Pathogen Host Interactions Database, http://www.phi-base.org/.

The NR database annotation showed that *Inonotus hispidus* and *Inocutis levis* both matched highly with *Sanghuangporus baumii* through 6,268 sequences (61.64%) and 6,287 sequences (62%) and that of *Fomitiporia mediterranea* through 2,042 sequences (20.08%) and 1,859 sequences (18.33%) (Suppl. materials 1, 2: fig. S2, tables S6, S7). Many genes annotated as “S: Function unknown” (2,709 in *Inonotus hispidus* and 2,552 in *Inocutis levis*) were found with the EggNOG database annotation (Suppl. materials 1, 2: fig. S3, table S8). The most frequent EggNOG category annotated in *Inonotus hispidus* was “O: Post-translational modification, protein turnover, chaperones” (567), while, in *Inocutis levis*, it was “L: Replication, recombination and repair” (952). In the KEGG database, both fungi had the highest annotation numbers in “Signal transduction” (622, 594) within “Environmental Information Processing” (Suppl. materials 1, 2: fig. S4, table S9), followed by “Carbohydrate metabolism” (415, 384), “Transport and catabolism” (348, 384), “Amino acid metabolism” (364, 343), “Cell growth and death” (352, 332) and “Translation” (341, 329). In the GO database, the annotations of two species were both divided into three classes with similar results (Suppl. materials 1, 2: fig. S5, table S10). The predicted genes were most related to biological process (5243, 5452), cellular nitrogen compound metabolic process (1703, 2097) and biosynthetic process (1462, 1906); cell (2559, 2942), intracellular (2470, 2862) and organelle (1941, 2357); molecular function (4943, 5151) and ion binding (2137, 2629). Amongst 10 databases, a total of 1,658 and 1,968 protein-coding genes were annotated in Cytochrome P450 database, which were identified with an E-value less than 1e^−5^ (Suppl. material 2: tables S11, S12).

**Figure 3.**
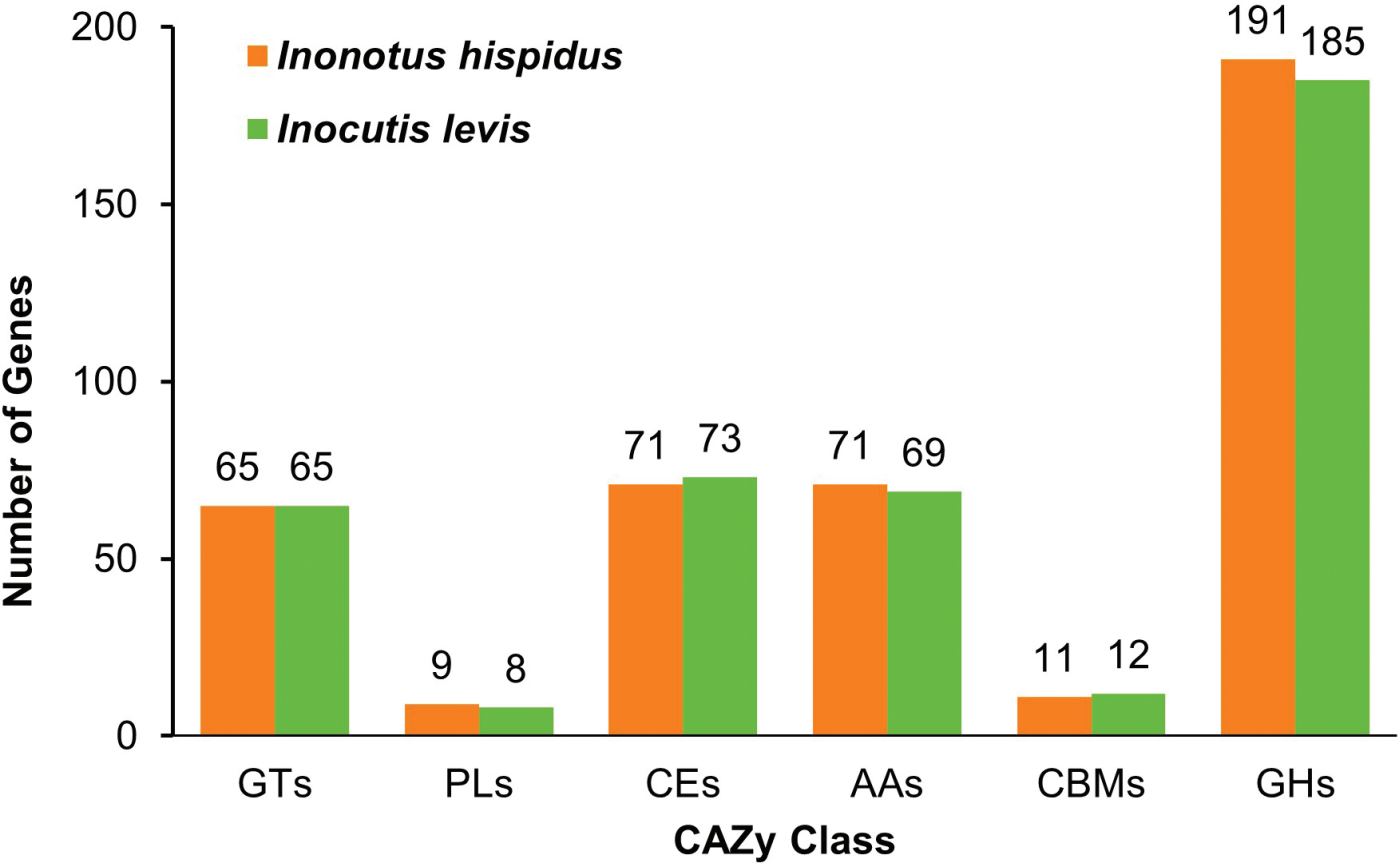
Annotation of *Inonotus hispidus* and *Inocutis levis* genes, based on CAZymes database. GTs: Glycosyl Transferases; PLs: Polysaccharide Lyases; CEs: Carbohydrate Esterases; AAs: Auxiliary Activities; CBMs: Carbohydrate-binding Modules; GHs: Glycoside Hydrolases.

*Inonotus hispidus* contained 418 genes related to CAZymes (412 in *Inocutis levis*), with a similar proportion across the six CAZymes categories. Glycoside Hydrolases (GHs) were most abundant in *Inonotus hispidus* (191) and *Inocutis levis* (185) (Fig. 3). Both species showed peak annotations in the CE10 (41 genes each), with abundant Auxiliary Activities (AAs), including AA9 (16, 15), AA2 (14, 15) and AA7 (13, 12, Suppl. material 2: tables S13, S14). Notably, the GH16 family (20) were annotated in *Inonotus hispidus*, while the GH18 family (41) were annotated in *Inocutis levis*.

**Figure 4.**
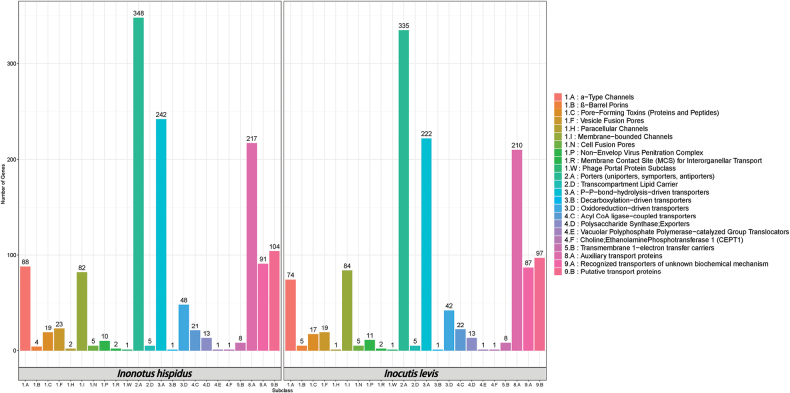
Annotation of *Inonotus hispidus* and *Inocutis levis*, based on TCDB database at the subclass level.

TCDB classifies membrane transport proteins into five hierarchical levels. At the class level (Suppl. material 2: tables S15, S16), both fungi had most annotations in “Electrochemical potential-driven transporters” (353 in *Inonotus hispidus* and 340 in *Inocutis levis*), followed by “Primary active transporters” (291, 265), “Channels/Pores” (236, 219), “Accessory factors involved in transport” (217, 210) and “Incompletely characterised transport systems” (195, 184). Fewer genes appeared in “Group translocators” (36, 37) and “Transmembrane electron carriers” (8, 8). At the subclass level (Fig. 4), “2.A: Porters (uniporters, symporters, antiporters)” contained most genes (348, 335). Additionally, the protein-coding genes of the two species were also annotated with protein subcellular localisation and secretion pathways, identifying 640 and 596 genes related to signal peptides, 1,688 and 1,632 genes related to transmembrane proteins and 433 and 398 genes related to secreted proteins (Suppl. material 2: tables S17, S18).

### ﻿Gene expression under PEG-induced drought stress

Sequencing the RNA of mycelia growing in PDA medium supplemented with 0% (CK), 10% (P10), 30% (P30) and 50% (P50) PEG-6000, yielded 90.15 Gb (*Inonotus hispidus*) and 89.39 Gb (*Inocutis levis*) raw data, with Q30 values exceeding 96% (Suppl. material 2: table S19). After quality control, 88.39 Gb and 87.39 Gb of clean data remained. These data were aligned to the assembled genomes, resulting in average alignment rates of 92.99% and 95.1% for the two species.

**Figure 5.**
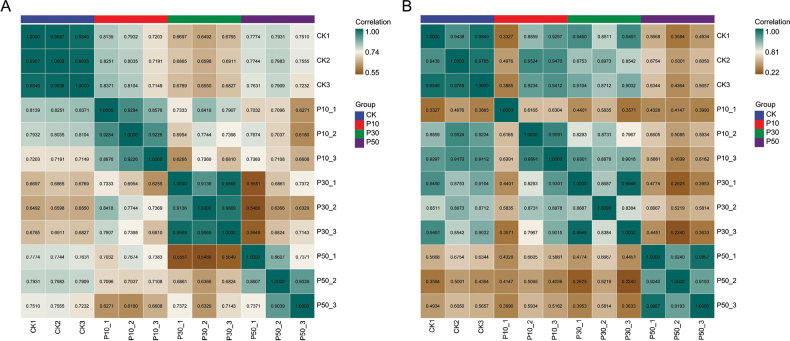
The correlation analyses within different concentrations of PEG-6000. A *Inonotus hispidus*; B *Inocutis levis*. CK: 0% PEG; P10: 10% PEG; P30: 30% PEG; P50: 50% PEG.

**Figure 6.**
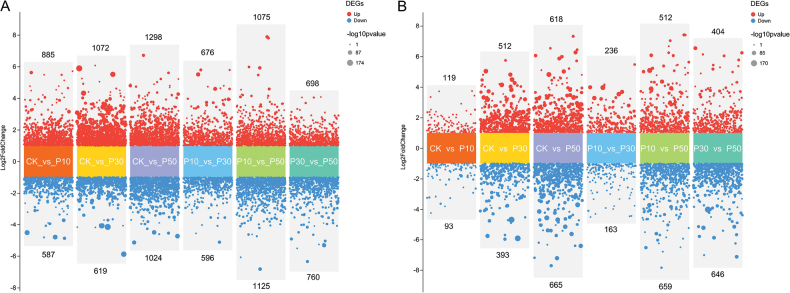
The number of DEGs amongst six comparison groups. A *Inonotus hispidus*; B *Inocutis levis*. The number of up- and down-regulated genes for each category are shown above and below the plot, respectively.

Prominent distinctions emerged amongst the three concentrations of PEG-6000 and control groups (Fig. 5). *Inonotus hispidus* exhibited more DEGs than *Inocutis levis* across all six pairwise comparisons, with totals of 4,550 and 2,113 DEGs, respectively (Fig. 6). Both up- and down-regulated genes progressively increased with drought intensity, peaking in CK_vs_P50 (2,322 vs. 1,283 DEGs). Most DEGs showed 2- to 4-fold expression changes, while a minority of genes exhibit changes exceeding 16-fold. Clustering analysis grouped all DEGs into five co-expression patterns (Suppl. material 1: fig. S6), where genes within clusters likely share regulatory mechanisms or functional relationships.

**Figure 7.**
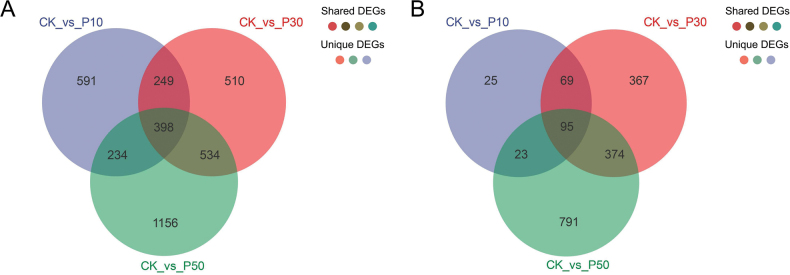
The shared and unique DEGs amongst three comparison combinations with different concentrations of PEG-6000. A *Inonotus hispidus*; B *Inocutis levis*.

Amongst the three concentrations of PEG-6000, 398 shared DEGs in *Inonotus hispidus* and 95 DEGs in *Inocutis levis* (Fig. 7). The CK_vs_P50 comparison showed the largest differential expression (1,156 and 791 DEGs). The top five most significantly up- and down-regulated genes across comparisons are detailed in Suppl. material 2: table S20.

### ﻿GO enrichment analyses

**Figure 8.**
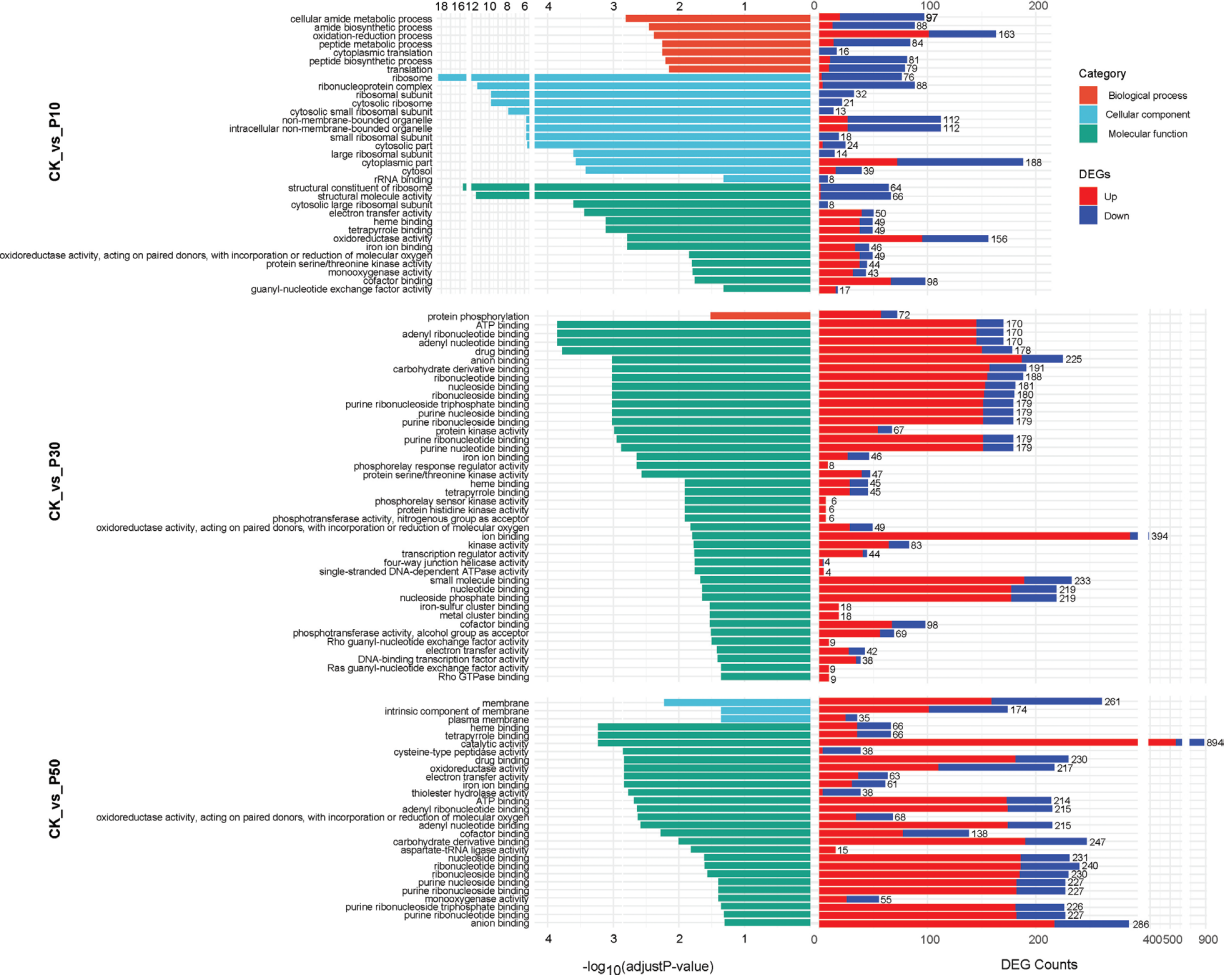
GO enrichment analyses of DEGs in *Inonotus hispidus* amongst three comparisons with different concentrations of PEG-6000. All terms with significant enrichment are shown.

**Figure 9.**
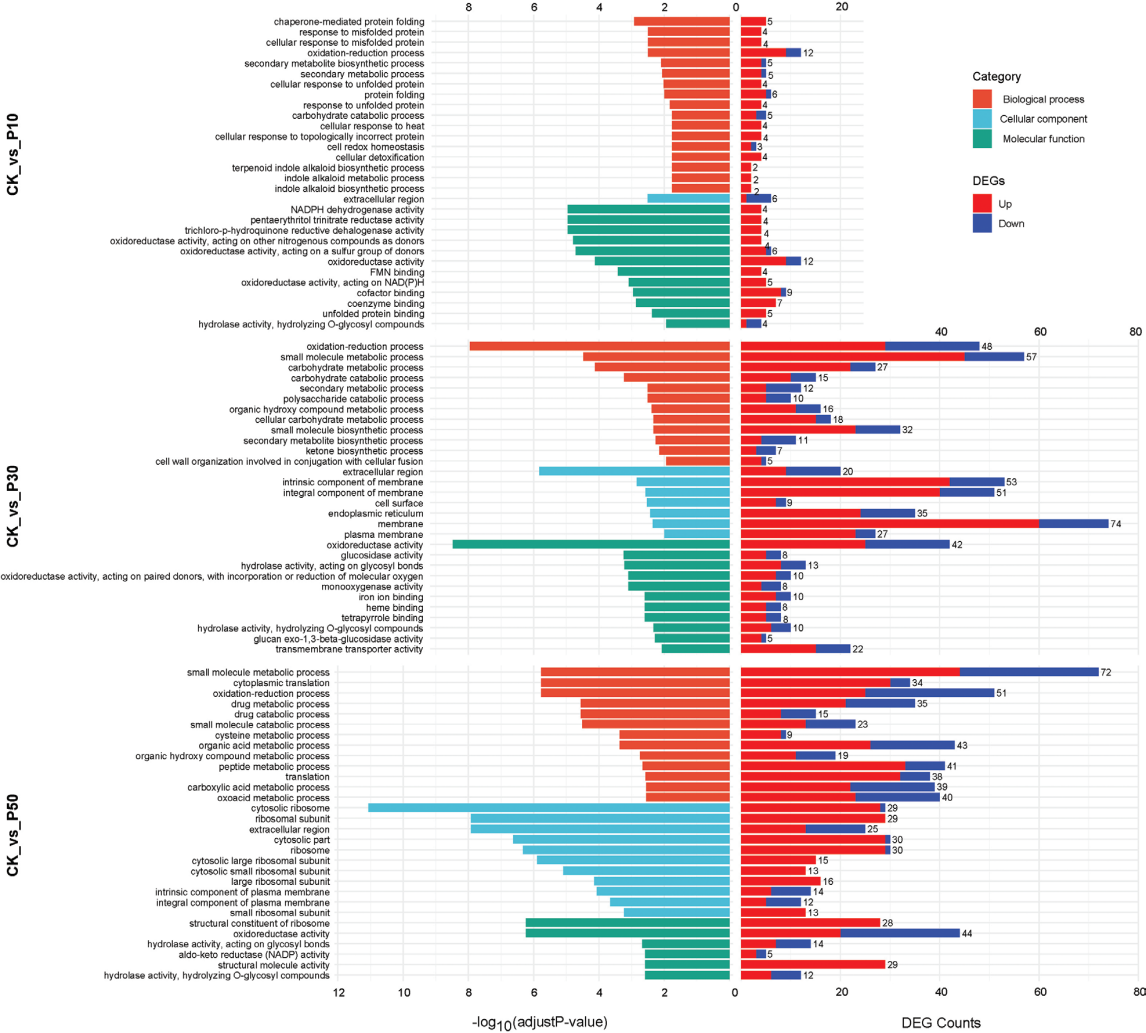
GO enrichment analyses of DEGs in *Inocutis levis* amongst three comparisons with different concentrations PEG. Top 30 significantly enriched terms are shown.

Under mild drought conditions (CK_vs_P10), the DEGs of *Inonotus hispidus* and *Inocutis levis* were enriched in 2,497 and 741 GO terms, respectively, with 33 and 55 significantly enriched terms (Figs 8, 9). *Inonotus hispidus* showed down-regulated DEGs with enrichment in ribosome-related terms, while up-regulated genes were enriched in redox, substance binding and signal transduction. *Inocutis levis* exhibited up-regulated DEGs with enrichment in oxidation-reduction, protein folding, stress responses and metabolism.

Under moderate drought conditions (CK_vs_P30), the DEGs of the two fungi were enriched in 2,589 and 2,751 GO terms, respectively, with 42 and 89 significantly enriched terms (Figs 8, 9). Up-regulated genes increased significantly, showing enhanced enrichment compared with CK_vs_P10 and they were further up-regulated in related items such as energy metabolism, molecular binding, protein phosphorylation, signal transduction and redox. In addition, *Inonotus hispidus* up-regulated DNA repair and transcriptional regulation terms, while *Inocutis levis* up-regulated carbohydrate metabolism, secondary metabolism and membrane transport mechanisms.

Under severe drought conditions (CK_vs_P50), the DEGs of the two fungi were enriched in 2,938 and 3,417 GO terms, respectively, with 28 and 76 significantly enriched terms, functionally similar to P30 responses (Figs 8, 9). *Inonotus hispidus* showed catalytic activity category (894 DEGs), with up-regulated genes far exceeding down-regulated genes, indicating enhanced enzymatic activity. Additionally, three new added entries in the cellular component category were related to membranes, suggesting further adjustments in membrane function to maintain intracellular homeostasis. *Inocutis levis* exhibited significant up-regulation of ribosome and translation-related processes and expanded organic acids and sulphur compounds.

### ﻿KEGG enrichment analyses

KEGG enrichment analyses identified the top five enriched pathways for two species (Table 4). Across three comparisons (CK_vs_P10, CK_vs_P30 and CK_vs_P50), the DEGs of *Inonotus hispidus* were significantly enriched in 1, 5 and 1 GO term, respectively. The DEGs of *Inocutis levis* had 0, 1 and 3 significantly enriched GO terms (Table 4). Under mild drought (CK_vs_P10), *Inonotus hispidus* exhibited significant enrichment in the “Ribosome” pathway, consisting entirely of down-regulated genes (64), while *Inocutis levis* displayed no significantly enriched pathways. Under moderate drought (CK_vs_P30), *Inonotus hispidus* exhibited significant up-regulation in pathways including Cell Cycle-Yeast, Meiosis-Yeast, ABC Transporters and DNA Replication. Its energy metabolism-related Methane Metabolism pathway showed relatively balanced up- and down-regulated genes, while *Inocutis levis* was only significantly enriched in the Pentose and Glucuronate Interconversions pathway. Under severe drought (CK_vs_P50), *Inonotus hispidus* was significantly enriched in the Other Glycan Degradation pathway, while *Inocutis levis* was significantly enriched in Starch and Sucrose Metabolism.

**Table 4.** KEGG enrichment analyses of *Inonotus hispidus* and *Inocutis levis*. The significantly enriched pathways of DEGs are shown.

Inonotus hispidus	Pathway ID	Pathway	Level 1	Up	Down	DEGs	Total	adjust-P
CK_vs_P10	ko03010	Ribosome	Genetic Information Processing	0	64	64	105	7.79E-24
CK_vs_P30	ko04111	Cell cycle-yeast	Cellular Processes	26	1	27	81	0.007
	ko04113	Meiosis-yeast	Cellular Processes	19	1	20	60	0.026
	ko02010	ABC transporters	Environmental Information Processing	9	0	9	18	0.026
	ko00680	Methane metabolism	Metabolism	5	4	9	20	0.049
	ko03030	DNA replication	Genetic Information Processing	11	0	11	28	0.049
CK_vs_P50	ko00511	Other glycan degradation	Metabolism	5	2	7	8	0.017
Inocutis levis	Pathway ID	Pathway	Level 1	Up	Down	DEGs	Total	adjust-P
CK_vs_P30	ko00040	Pentose and glucuronate interconversions	Metabolism	5	3	8	23	0.003
CK_vs_P50	ko03010	Ribosome	Genetic Information Processing	28	0	28	103	2.47E-05
	ko00460	Cyanoamino acid metabolism	Metabolism	6	1	7	16	0.016
	ko00500	Starch and sucrose metabolism	Metabolism	8	5	13	48	0.016

### ﻿Expression levels of genes involved in responses to drought stress

Integrating GO and KEGG functional enrichment analyses, with other significantly altered or highly expressed genes and based on functional annotation results, the DEGs in *Inonotus hispidus* and *Inocutis levis* that respond to drought stress were filtered by P-value < 0.05 and |log2Foldchange| > 1 and classified into four categories in this study: antioxidant-related genes, osmoregulation genes, signal transduction genes and ribosomal genes.

#### ﻿Antioxidant-related genes (Suppl. material 1: fig. S7)

Amongst antioxidant enzymes-related DEGs, *Inonotus hispidus* contained three superoxide dismutase (*SOD*), two catalase (*CAT*), 22 peroxidase (*POD*) and five thioredoxins (*Trx*) genes, while *Inocutis levis* had one *SOD*, one *CAT*, 15 *POD* and seven Trx genes. Both species exhibited 21 and 22 DEGs related to glutathione, predominantly glutathione S-transferases (*GST*). Ascorbic acid (vitamin C) as non-enzymatic antioxidants contributes significantly to biological antioxidant defence (Agwu et al. 2023). The “Ascorbate and aldarate metabolism” pathway contained 14 in *Inonotus hispidus* and four in *Inocutis levis*, involved in ascorbic acid synthesis. *Inonotus hispidus* shows significant enrichment in “Other glycan degradation” (7 DEGs mainly related to N-glycan) and “Mannose type O-glycan biosynthesis” (4 DEGs). *Inocutis levis* exhibited enrichment in four polysaccharide metabolism (16 DEGs) and seven DEGs in the “Cyanoamino acid metabolism”, where some of the hydrolysis products might participate in antioxidant-related pathways in subsequent metabolic processes.

#### ﻿Osmoregulation genes (Suppl. material 1: fig. S8)

*Inonotus hispidus* and *Inocutis levis* exhibited three and one trehalose-related DEGs, respectively. Both fungi showed DEGs enrichment in the “Fructose and mannose metabolism” pathway (13 DEGs in *Inonotus hispidus* and 5 DEGs in *Inocutis levis*) (Suppl. material 1: fig. S8). Notably, *Inonotus hispidus* expressed a DEG (scaffold8.g95) within the significantly enriched “Pentose and glucuronate interconversions” pathway, annotated as L-iditol 2-dehydrogenase. This pathway was associated with carbon source utilisation and cell wall re-modelling. *Inocutis levis* showed significant enrichment of 13 DEGs in “Starch and sucrose metabolism”, with predominantly up-regulation expression.

*Inonotus hispidus* exhibited four DEGs related to proline metabolism compared to the one DEG of *Inocutis levis*, including one proline dehydrogenase gene. With the deepening of drought stress, expression analysis revealed down-regulation of this gene in *Inonotus hispidus*, while *Inocutis levis* showed up-regulation and down-regulation.

The two fungi showed significant enrichment of DEGs associated with transmembrane transport proteins, critical for drought adaption by regulating intracellular and extracellular substances exchange. *Inonotus hispidus* showed significant enrichment in the “ABC transporters” pathway, with nine DEGs all up-regulated during P30. *Inocutis levis* similarly displayed prominent up-regulation of 22 transmembrane transport-related DEGs at P30, suggesting shared drought adaption strategies through the transport protein pathway. Both fungi had one DEG annotated as aquaporin-9, which was progressively down-regulated with increasing stress. Additionally, in *Inocutis levis*, expression of an aquaglyceroporin-related gene was down-regulated in mild/moderate drought conditions and up-regulated in severe drought conditions. Unlike conventional aquaporins, both proteins facilitate transport of small molecules like glycerol, contributing to the osmotic environment adaptation.

#### ﻿Signal transduction genes (Suppl. material 1: fig. S9)

Both fungi exhibited 15 DEGs in the “yeast MAPK signalling pathway” (Suppl. material 1: fig. S9), including up-regulated guanine nucleotide exchange factors (GEFs): one DEG “GDP-GTP exchange protein 1 in *Inonotus hispidus* and three DEG “GDP-GTP exchange protein 2 in *Inocutis levis*.

Under moderate drought (CK_vs_P30), *Inonotus hispidus* showed significant GO enrichment for guanylate nucleotides (four entries), comprising a total of 20 DEGs, 18 of which are up-regulated. *Inocutis levis* exhibited enrichment across comparisons for “response to heat” and “response to temperature stimulus” (14 DEGs) including two MAPK-related genes. Amongst its 11 up-regulated DEGs, six encoded heat shock proteins (HSPs) were present.

#### ﻿Ribosomal genes (Fig. 10)

**Figure 10.**
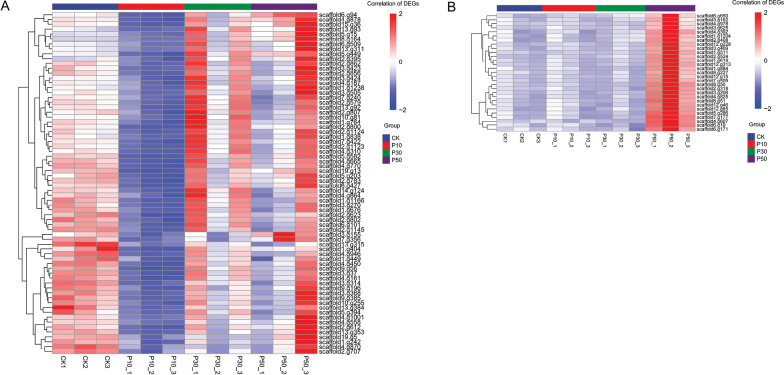
Significant Ribosomal DEGs that may be related to drought stress. A *Inonotus hispidus*; B *Inocutis levis*. Genes with similar expression patterns were clustered together. Colour scale represents the correlation from red to blue.

Ribosomes primarily facilitate protein synthesis. GO and KEGG annotations identified DEGs in ribosome pathway, revealing contrasting expression patterns between species under drought stress (Fig. 10). *Inonotus hispidus* exhibited 70 significantly down-regulated ribosome DEGs during mild drought, while *Inocutis levis* had 29 significantly up-regulated ribosomal DEGs under severe drought. This marked contrast may reflect distinct adaptive strategies: it is possible that *Inonotus hispidus* initially suppresses ribosomal genes expression to conserve energy during mild stress, subsequently up-regulating these genes as conditions intensify. Conversely, *Inocutis levis* up-regulates ribosomal genes during severe drought, suggesting a mechanism to sustain cellular function under extreme osmotic challenge.

## ﻿Discussion

*Inonotus hispidus* and *Inocutis levis* are some of the few wood-inhabiting fungi that naturally grow on *Populus euphratica* in the field (Qiao et al. 2008), indicating their potential adaptation to extreme drought conditions. However, the genes and mechanisms at the molecular level involved in their drought tolerance regulation remain unclear. In this study, we performed the whole-genome sequencing of two strains of *Inonotus hispidus* and *Inocutis levis* growing on *P. euphratica* in Xinjiang, China. The genomes were assembled to 34.57 Mb and 37.17 Mb, respectively. Currently, three whole-genome sequences of *Inonotus hispidus* have been reported, collected separately from mulberry trees in Linyi, Shandong (35.69 Mb) (Tang et al. 2022) and Xiajin County, Shandong (34.14 Mb) (Wang et al. 2023) and from southern Xinjiang (34.02 Mb) (Zhang et al. 2022). The genomes of two other species of *Inonotus* are also available, two of *Inonotus obliquus* (Ach.ex Pers.) Pilát genomes (38.18 Mb and 36.13 Mb) (Duan et al. 2022; Hao et al. 2023) and one of *Inonotus vitis* A.A. Brown, D.P. Lawr. & K. Baumgartner (35.3 Mb) (Garcia et al. 2024). However, no whole-genome data were available for *Inocutis*. The two genera belong to *Hymenochaetales*, which have 31 published genomes ranging from 28.6 Mb to 150.86 Mb and GC% ranging from 40.83% to 52.43% (Zhao et al. 2024). In this study, the assembled genome sizes were within the expected range and they possessed high BUSCO scores (97.30%, 96.60%), indicating that the assembled genomes were of high quality to facilitate subsequent omics analyses.

Our comparative genomic analysis revealed the genomes of the two fungi displayed a remarkable level of similarity and evolutionary conservation (Fig. 2), sharing 7,226 homologous genes and exhibiting 69.61% collinear regions. High collinearity within a single species generally implies a relatively stable arrangement of chromosomal segments during evolution and preservation of functional connections between genes (Sasaki 2005; Tang et al. 2008). This conservation was also reflected in phylogenetic relationships (Wu et al. 2022). Despite these evolutionary similarities, subtle differences were observed, particularly in gene duplications and structural re-arrangements. *Inocutis levis* exhibited higher genome-wide collinearity (Fig. 1), with more repetitive sequences (4.76%) and non-coding RNAs (0.46%) than *Inonotus hispidus* (2.10%, 0.12%) (Suppl. material 2: tables S4, S5), potentially reflecting distinct adaptive strategies. Accumulation of repetitive sequences is usually associated with active transposon activity or recent bursts. Generally, since transposon activity disrupts collinearity, an increase in duplicate genes is often associated with low collinearity (Bourque et al. 2018). In this study, *Inocutis levis* maintained high collinearity while accumulating repetitive sequences. This may occur because its repetitive sequences tend to be inserted into intergenic regions or introns (non-coding regions) (Singleton and Levin 2002); collinearity is retained by suppressing transposon activity through epigenetic regulation (such as DNA methylation) (Slotkin and Martienssen 2007); or the accumulation of repetitive sequences occurred relatively shortly after species divergence and has not yet disrupted gene arrangement (Gaiero et al. 2019). In contrast, *Inonotus hispidus* had a lower synonymous substitution rate, was more conserved and displayed less genomic collinearity and fewer repetitive sequences. This may result from more chromosomal structural variations (Dhakal et al. 2024) or purifying selection in natural selection (Okagaki et al. 2016). The specific reasons require further verification through genomic structure analysis (such as Hi-C technology) and transposon activity assessment. In addition, genomic repetitive sequences are generally regarded as an important evolutionary driver (Biscotti et al. 2015), aiding rapid adaptation under stress conditions and the evolution of novel genes with host-beneficial functions (Mehrotra and Goyal 2014).

The impact of drought stress on organisms is multifaceted and complex. We observed that a 50% concentration (P50) severely inhibited mycelial growth, coinciding with peak differential gene expression (*Inonotus hispidus*: 2,322 DEGs, *Inocutis levis*: 1,283 DEGs) (Fig. 3). The consistency between phenotypic responses and transcriptomic profiles validated P50 as the critical stress threshold. Combining genome functional annotation with transcriptome analysis indicated that the two fungi employ multi-level molecular mechanisms to operate their response to drought stress together.

Drought stress induces oxidative stress in organisms, triggering antioxidant defence systems to eliminate reactive oxygen species (*ROS*) (Das and Roychoudhury 2014). These defences typically involve both enzymatic antioxidants (e.g. *SOD*, *CAT*, *POD*) and non-enzymatic compounds (Das and Roychoudhury 2014). Glutathione, a potent antioxidant, cycles between reduced (*GSH*) and oxidised (*GSSG*) forms to mitigate *ROS* (Zhang et al. 2020; Liu et al. 2022). Under increasing drought stress, both fungi up-regulated antioxidant-related genes, such as *SOD*, *CAT*, ascorbate and polysaccharides (Suppl. material 1: fig. S7). This response reduced *ROS* accumulation, minimised membrane lipid peroxidation and facilitated DNA damage repair (Fujita and Hasanuzzaman 2022). Notably, polysaccharide-related DEGs were enriched (primarily upregulated), aligning with their established antioxidant function in mushrooms (Chun et al. 2021) and prior isolation of bioactive polysaccharides of these fungi (Vinogradov and Wasser 2005; Liu et al. 2019a, 2024).

Organisms synthesise intracellular osmolytes to counteract environmental stresses. Key osmotic regulators comprise soluble sugar (sucrose, fructose, trehalose and mannose) amino acids, like proline and glycine (Seleiman et al. 2021), amines such as betaine and polyamine and inorganic ions like K^+^, Ca^2+^ and Na^+^ (Wu et al. 2013). Under drought stress, both *Inonotus hispidus* and *Inocutis levis* appear to accumulate osmolytes (trehalose, fructose, sucrose, proline) to maintain water balance. Trehalose served as the primary fungal osmoprotectant (Wang et al. 2015), functioning through water replacement, entrapment and vitrification (Kuczyńska-Wiśnik et al. 2024), with drought-responsive trehalose-related DEGs identified (Suppl. material 1: fig. S8). Under severe drought, *Inocutis levis* mostly up-regulated genes in soluble sugar pathways (starch and sucrose metabolism), perhaps suggesting great reliance on sugars for osmo-protection and energy, while *Inonotus hispidus* perhaps suppressed sugar metabolism to conserve energy.

Proline represents one of the most stress-responsive amino acids in plants (Furlan et al. 2020), while its direct role in fungal drought stress remains unclear. During drought stress, plant cells accumulate proline and other osmolytes to maintain intracellular osmotic pressure and prevent excessive water loss (Das and Sarkar 2024). Both fungi differentially expressed a gene annotated as proline dehydrogenase and its low expression under moderate and severe drought suggested reduced proline decomposition, thereby maintaining intracellular proline accumulation, enhancing cell osmotic adjustment and drought tolerance. *Inonotus hispidus* up-regulated the ABC transporter pathway during moderate drought (P30) to facilitate osmo-protectant shuttling. In *Inocutis levis*, GO enrichment identified 22 DEGs in transmembrane transport-related terms (Suppl. material 1: fig. S8) maintaining ion homeostasis and antioxidant transport. Aquaporins (AQPs), located in the cell membrane, are specialised in water transport (Yu et al. 2024). The gene annotated as aquaporin-9 (AQP9) was down-regulated in both fungi with increasing drought stress. AQP9 is a glycerol-channel aquaporin membrane protein that has been shown to conduct water, glycerol as well as small solutes like urea, mannitol, sorbitol and uracil (Hibuse et al. 2006).

The MAPK (Mitogen-Activated Protein Kinase) pathway is a conserved fungal signal transduction system, responsible for regulating stress responses, growth, development and pathogenicity (Martínez-Soto and Ruiz-Herrera 2017). This cascade mediates adaptation to abiotic stresses including drought, temperature shifts and salinity (Hamel et al. 2012; Zhu 2016), with orthologues identified across 231 fungal species (Xu et al. 2017). This enables fungi to effectively adapt and survive under changing environmental conditions. Under moderate drought (P30), *Inonotus hispidus* primarily up-regulated MAPK pathway components, whereas *Inocutis levis* showed inconsistent expression patterns (Suppl. material 1: fig. S9). These results suggest that, despite broad conservation of this classical signalling pathway, species may exhibit regulatory differences when responding to identical stresses. Mechanistically, guanine nucleotide exchange factors (GEFs) activate small GTPases to promote signalling (Lu et al. 2016). Within MAPK signalling, heat shock proteins (HSPs) may modulate MAPK efficiency by maintaining protein folding integrity under stress (Piper et al. 2006; Verghese et al. 2012; Zhu et al. 2023), though direct interactions require further validation.

Notably, ribosomal genes expression differed markedly between the two fungi (Fig. 10). *Inonotus hispidus* significantly down-regulated ribosome-related genes under mild drought, with expression up-regulating as stress intensified. This pattern suggests resource conservation by suppressing protein synthesis and reducing translational energy expenditure, indicating prioritisation of metabolic regulation for energy allocation and physiological stability during mild stress. Conversely, *Inocutis levis* exhibited significant up-regulation of ribosome-related DEGs under severe drought, potentially enhancing protein synthesis to improve adaptability, bolster biosynthetic capacity and maintain cellular integrity. Ribosomal gene regulation in plants varies substantially with environmental changes like drought stress and light control (Lei et al. 2015; Moin et al. 2017; Shiraku et al. 2021; Hou et al. 2024). In some filamentous fungi, ribosomal gene down-regulation represents a conserved energy conservation strategy under mild-moderate drought stress and this repression, mediated through conserved signalling pathways, then redirected resources towards stress protectant synthesis (Petibon et al. 2020). Notably, some species exhibit a biphasic response: initial down-regulation under mild stress followed by re-activation during severe drought to support adaptive structure production or survival proteins, demonstrating a dynamic shift from general suppression to targeted adaptation (Garch et al. 2017). Deeper understanding of this expression pattern could elucidate fungal adaptive mechanisms to abiotic stresses, providing a theoretical basis for future genetic improvement of stress tolerance.

This study presents the first genome assembly of *Inocutis levis* and provides preliminary insights into drought adaptation mechanisms in both *Inonotus hispidus* and *Inocutis levis*. However, validation of key pathways requires multi-omics integration (e.g. proteomics, metabolomics). Functional assays (e.g. gene editing) could clarify candidate gene roles. *Inonotus hispidus* and *Inocutis levis*, as white-rot fungi in arid ecosystems, both hold potential for ecological restoration and stress-tolerant microbial resource development and that their lignocellulose decomposition capability enhances soil organic matter accumulation in degraded arid lands and the secreted metabolites (e.g. osmoprotectants, antioxidants) ameliorate abiotic stress in co-planted vegetation (Martínez et al. 2005). This warrants further exploration in several directions, such as metabolite profiling and consortium optimisation with the host.

## ﻿Conclusions

This study completed genome and transcriptome sequencing of two macrofungi, *Inonotus hispidus* and *Inocutis levis*, isolated from *Populus euphratica* under drought stress. The genome information was enriched with high-quality added genome data and the whole-genome map of *Inocutis levis* was developed for the first time. The structural and functional features of these genomes showed high similarity. Through *in vitro* experiments simulating three levels of drought stress, abundant DEGs were identified in the two fungi, which were involved in important pathways such as antioxidant defence, osmotic regulation, signal transduction and ribosomal function regulation. *Inonotus hispidus* and *Inocutis levis* shared comparable antioxidant enzyme gene expression patterns, but employed divergent ribosomal strategies: *Inonotus* hispidus suppressed protein synthesis to conserve energy during initial drought, whereas *Inocutis levis* upregulated it under prolonged stress to bolster adaptive capacity. The results of this study provide valuable data supporting the understanding of molecular adaptation mechanisms in fungi under drought conditions, highlighting their potential in drought stress research.

## ﻿Additional information

### Use of AI

No use of AI was reported.

### Adherence to national and international regulations

All the fungal strains used in this study have been legally obtained, respecting the Convention on Biological Diversity (Rio Convention).

### Author ORCIDs

Miao Zhou https://orcid.org/0009-0001-7137-4929

Yi-Fei Sun https://orcid.org/0000-0003-3997-3662

Bao-Kai Cui https://orcid.org/0000-0003-3059-9344

### Data availability

All data generated or analysed for this study are included in this article and its supplementary files. The raw sequencing data of the genome and transcriptome have been deposited in NCBI under the BioProject ID PRJNA1209923. The genome accessions are JBKOSQ000000000 for *Inonotus hispidus* Wu 2022-1 and JBKOSR000000000 for *Inocutis levis* Cui 19065, respectively.

## Supplementary materials

Supplementary material 1 Supplementary images Data type pdf Explanation note **figure S1.** Distribution of K-mer depth frequency in genome scope profile. **figure S2.** The homologous species distribution of NR at the genus level. **figure S3.** Annotation of *Inonotus hispidus* and *Inocutis levis*, based on EggNOG database. **figure S4.** Annotation of *Inonotus hispidus* and *Inocutis levis*, based on KEGG database. **figure S5.** Annotation of *Inonotus hispidus* and *Inocutis levis*, based on GO database. **figure S6.** Cluster diagram of DEGs. **figure S7.** Antioxidant-related DEGs that might be related to the drought stress. **figure S8.** Osmoregulation DEGs that might be related to the drought stress. **figure S9.** Signal transduction DEGs that might be related to the drought stress. https://binary.pensoft.net/file/1416790This dataset is made available under the Open Database License (http://opendatacommons.org/licenses/odbl/1.0/). The Open Database License (ODbL) is a license agreement intended to allow users to freely share, modify, and use this Dataset while maintaining this same freedom for others, provided that the original source and author(s) are credited.Miao Zhou, Meng-Xue Lv, Dong-Mei Wu, Neng Gao, Tai-Min Xu, Yi-Fei Sun, Bao-Kai Cui

Supplementary material 2 Supplementary tables Data type xlsx Explanation note The following supporting information can be downloaded at https://. **table S1.** Statistics of Illumina NovaSeq sequencing data volume information. **table S2.** Statistics of pacbio Sequel sequencing data volume information. **table S3.** K-mer=19 analysis results for genomic sequencing of two fungi. **table S4.** Classification statistics of repeated sequences for two fungi. **table S5.** Classification statistics of non-coding RNA for two fungi. **table S6.** Annotation of *Inonotus hispidus*, based on NR database. **table S7.** Annotation of *Inocutis levis*, based on NR database. **table S8.** Annotation of two fungi on EggNOG database. **table S9.** Annotation of two fungi on KEGG database. **table S10.** Annotation of two fungi on GO database. **table S11.** Annotation of *Inonotus hispidus*, based on P450 database. **table S12.** Annotation of *Inocutis levis*, based on P450 database. **table S13.** Annotation of *Inonotus hispidus*, based on CAZymes database. **table S14.** Annotation of *Inocutis levis*, based on CAZymes database. **table S15.** Annotation of *Inonotus hispidus*, based on TCDB database. **table S16.** Annotation of *Inocutis levis*, based on TCDB database. **table S17.** Signal peptides prediction for two fungi. **table S18.** Transmembrane proteins prediction for two fungi. **table S19.** Summary of the RNA-seq data utilised in this study. **table S20.** Top 5 DEGs in each group of two fungi. https://binary.pensoft.net/file/1416791This dataset is made available under the Open Database License (http://opendatacommons.org/licenses/odbl/1.0/). The Open Database License (ODbL) is a license agreement intended to allow users to freely share, modify, and use this Dataset while maintaining this same freedom for others, provided that the original source and author(s) are credited.Miao Zhou, Meng-Xue Lv, Dong-Mei Wu, Neng Gao, Tai-Min Xu, Yi-Fei Sun, Bao-Kai Cui
