# Supplementary material for: ﻿Drought stress responses revealed by genomic and transcriptomic analyses of two macrofungi (Inonotus hispidus and Inocutis levis) from Populus euphratica
Source: IMA Fungus. 2025 Sep 15;16:e163859. doi: 10.3897/imafungus.16.163859 (PMC12455214; doi:10.3897/imafungus.16.163859)
Supplement: Supplementary material 1 — Supplementary images [file imafungus-16-e163859-s001.pdf]

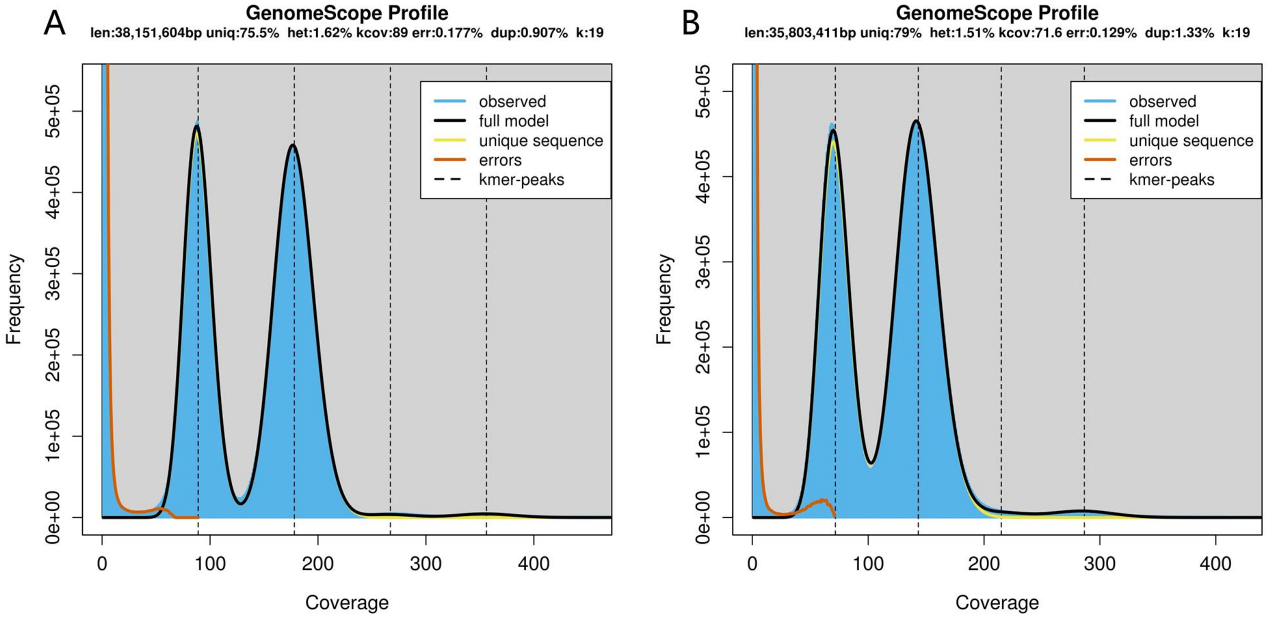

Figure S1. Distribution of K-mer depth frequency in genome scope profile. (A) *Inonotus hispidus*, (B) *Inocutis levis*.

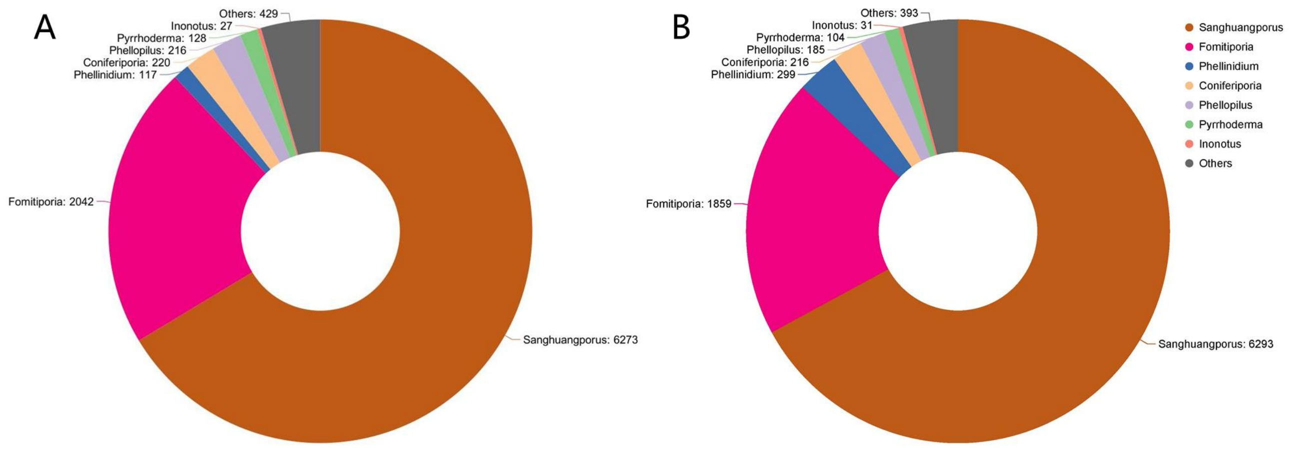

Figure S2. The homologous species distribution of NR at the genus level. (A) *Inonotus hispidus*, (B) *Inocutis levis*.

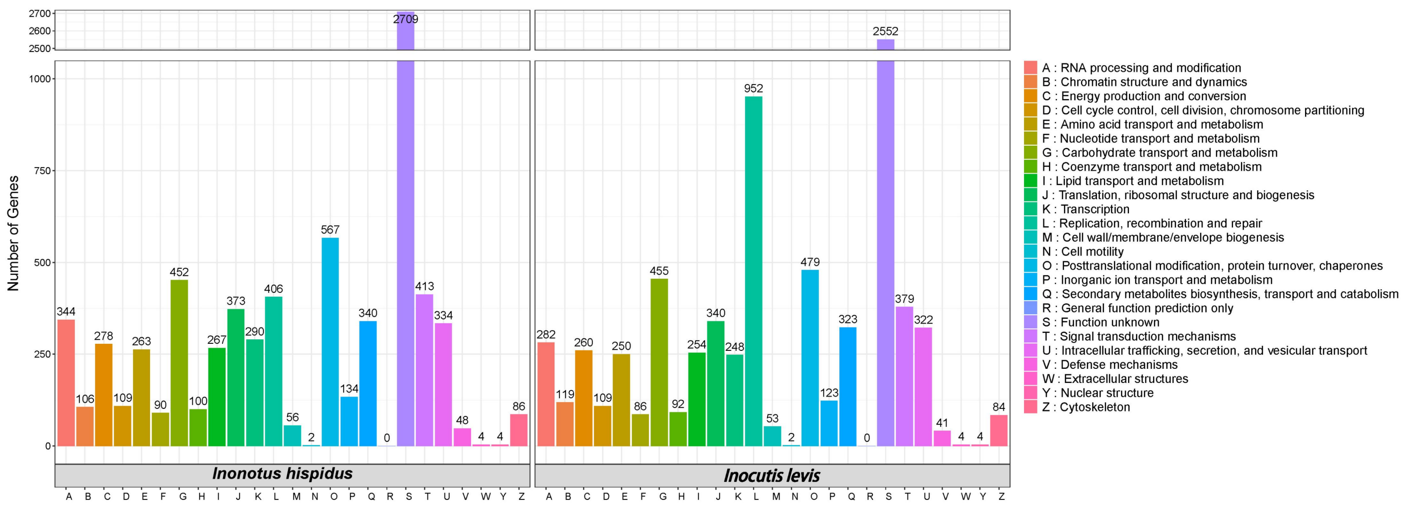

Figure S3. Annotation of *Inonotus hispidus* and *Inocutis levis* based on EggNOG database.

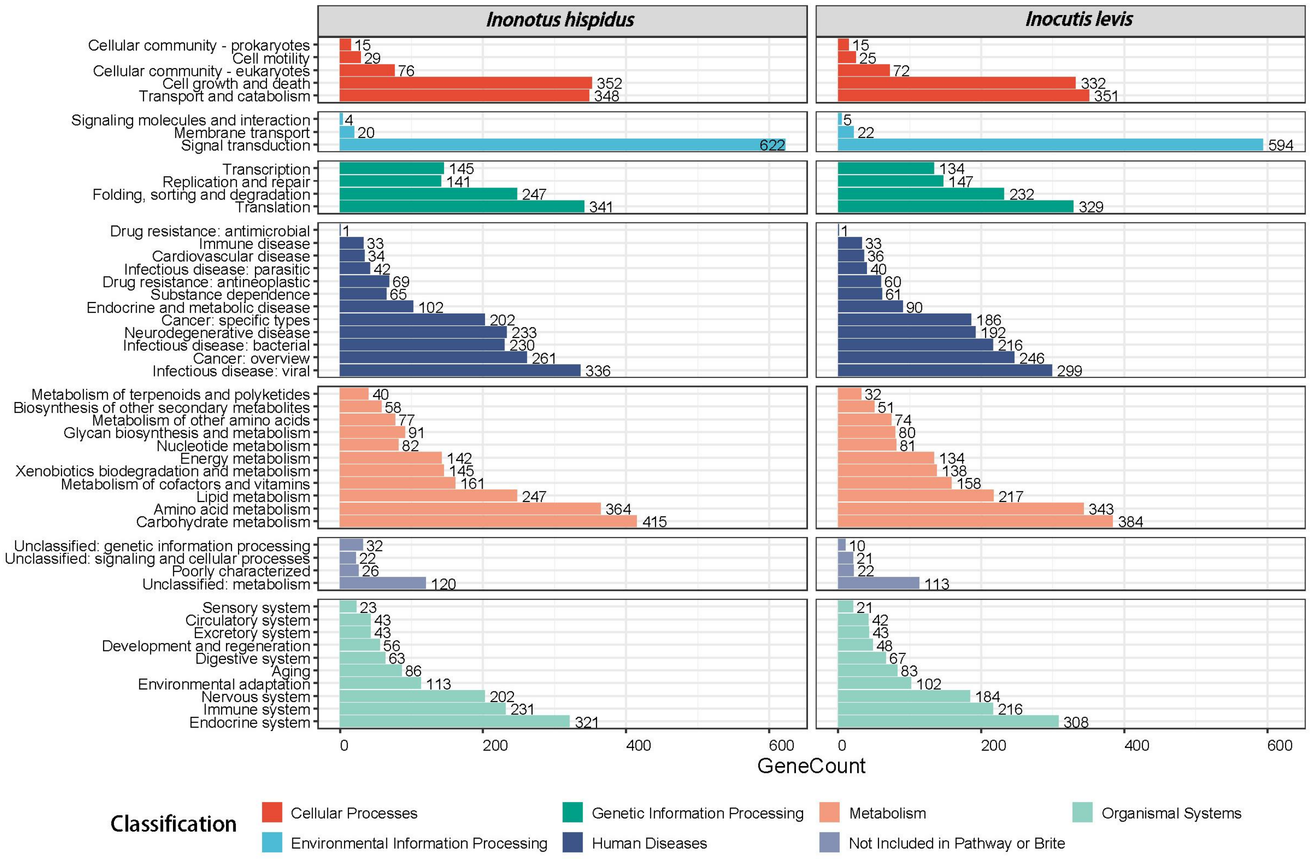

Figure S4. Annotation of *Inonotus hispidus* and *Inocutis levis* based on KEGG database.

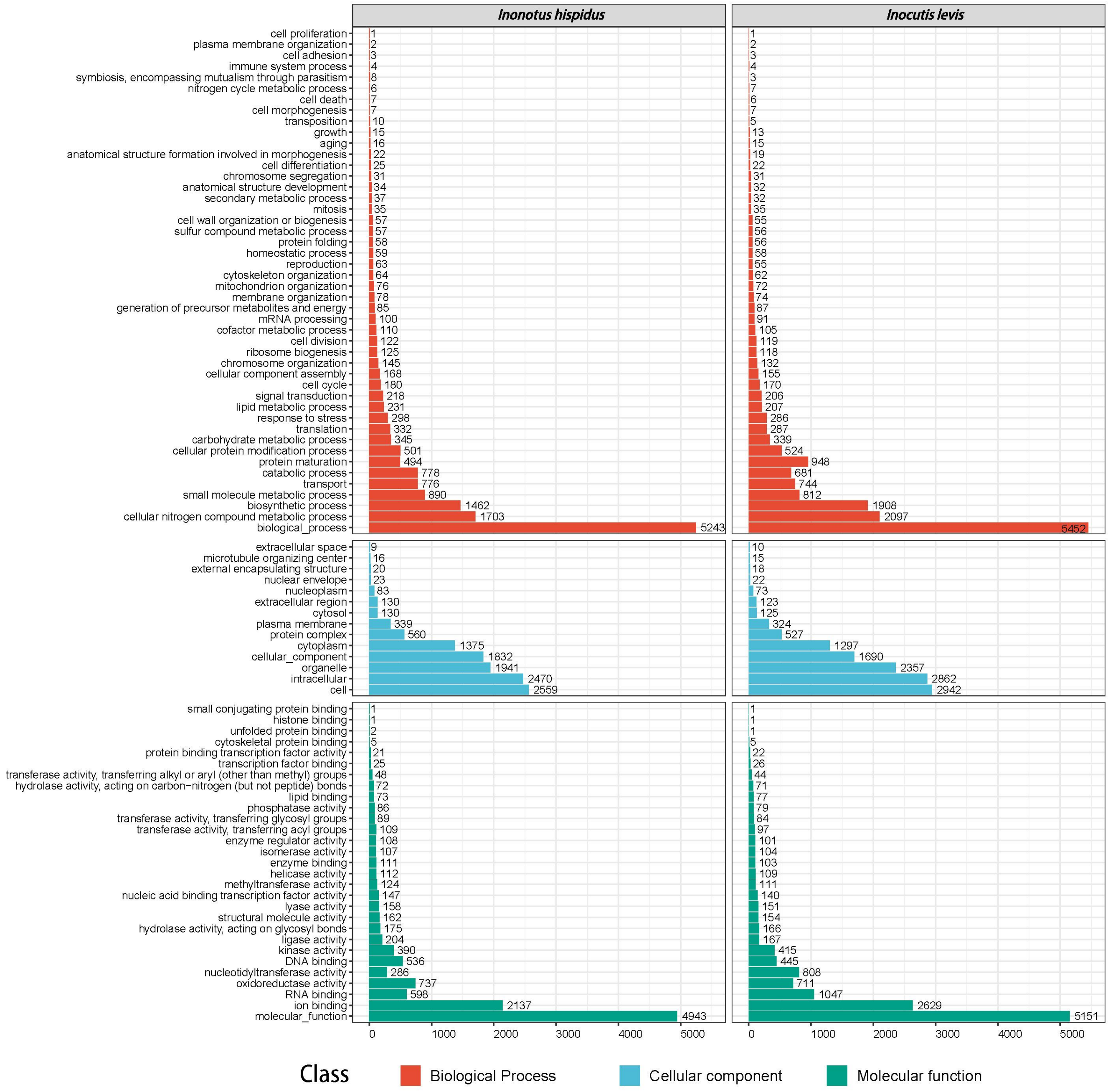

Figure S5. Annotation of *Inonotus hispidus* and *Inocutis levis* based on GO database.

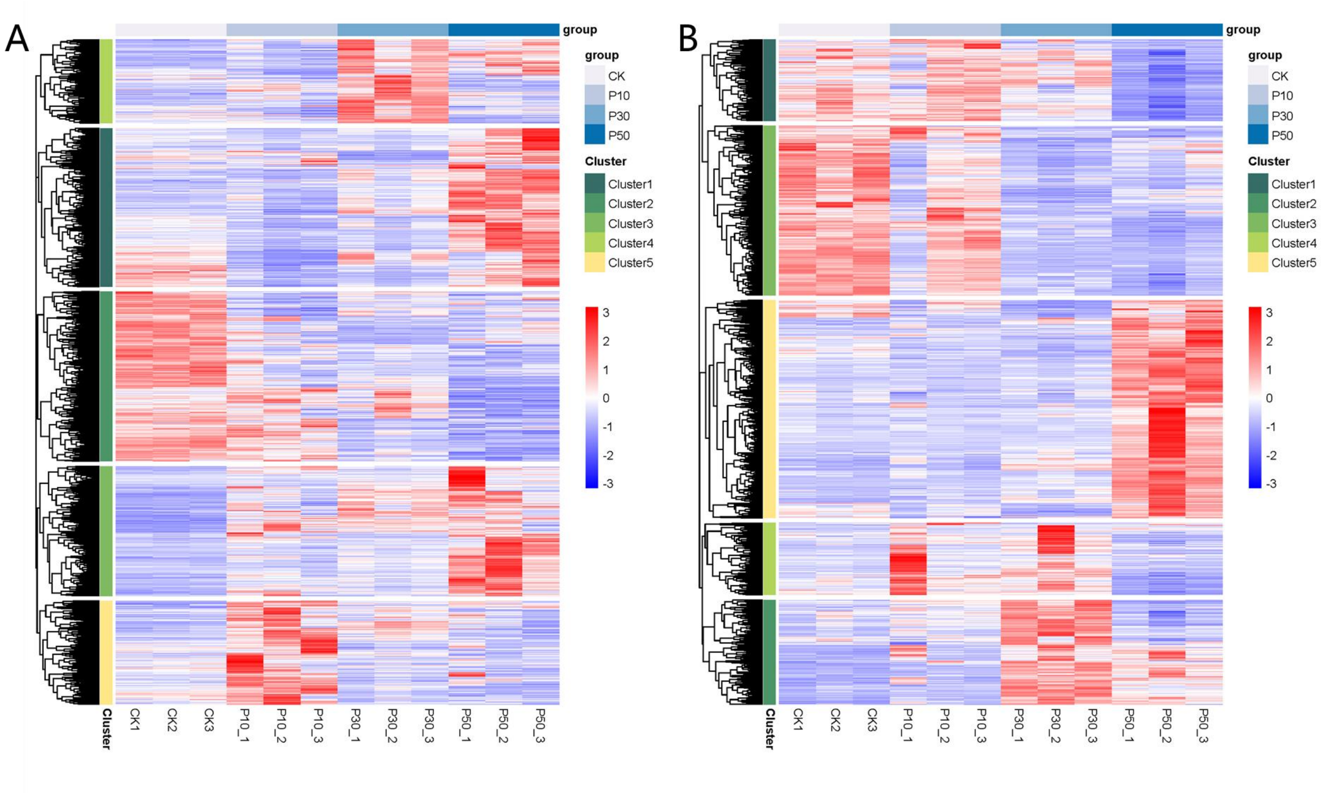

Figure S6. Cluster diagram of DEGs. (A) *Inonotus hispidus*, (B) *Inocutis levis*. Each column represents a sample, with genes indicated horizontally. All DEGs were clustered into five groups, where genes exhibiting high expression correlation across samples were grouped together. The different colors of the heatmap, ranging from blue over white to red, represent scaled expression levels of genes with the FPKM values after Z-score normalization.

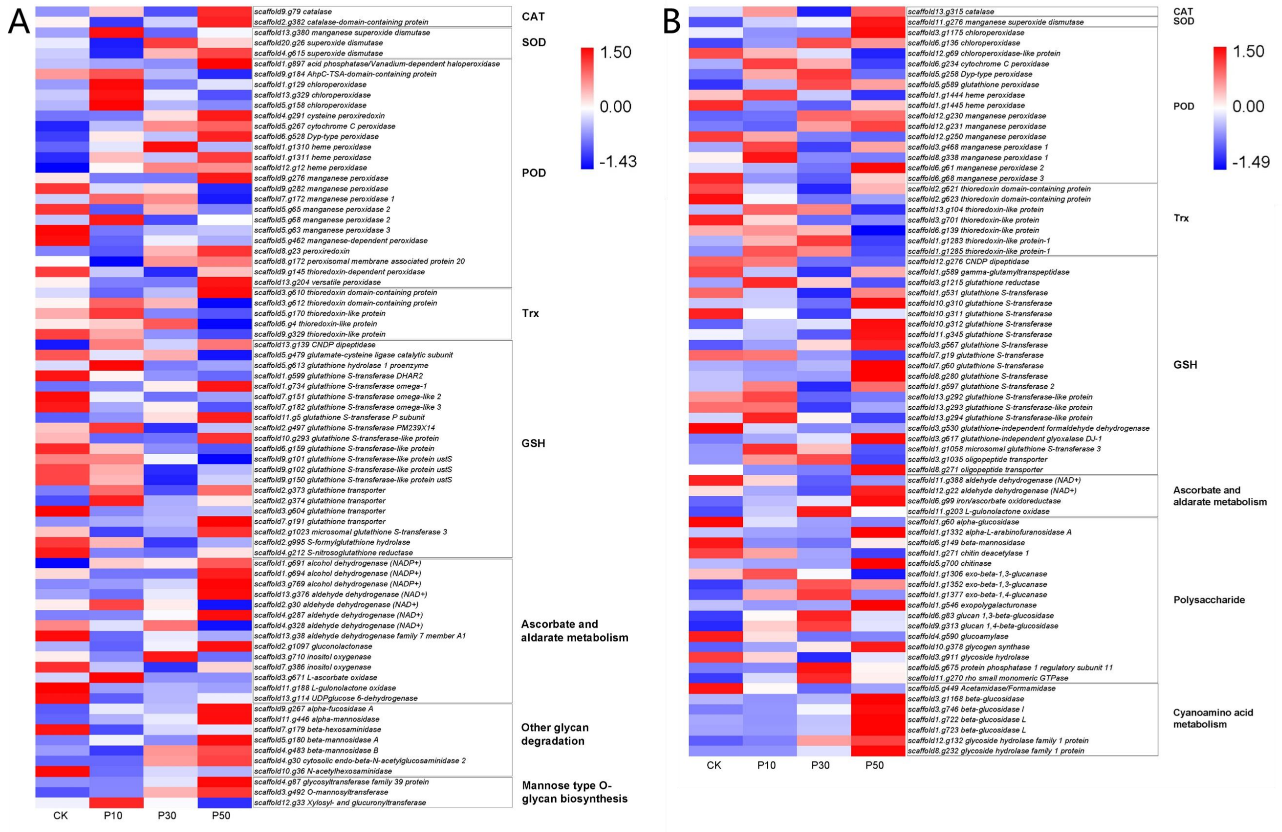

Figure S7. Antioxidant - related DEGs that might be related to the drought stress. (A) *Inonotus hispidus*, (B) *Inocutis levis*. The different colors of the heatmap, ranging from blue over white to red, represent scaled expression levels of genes with the FPKM values after Z-score normalization.

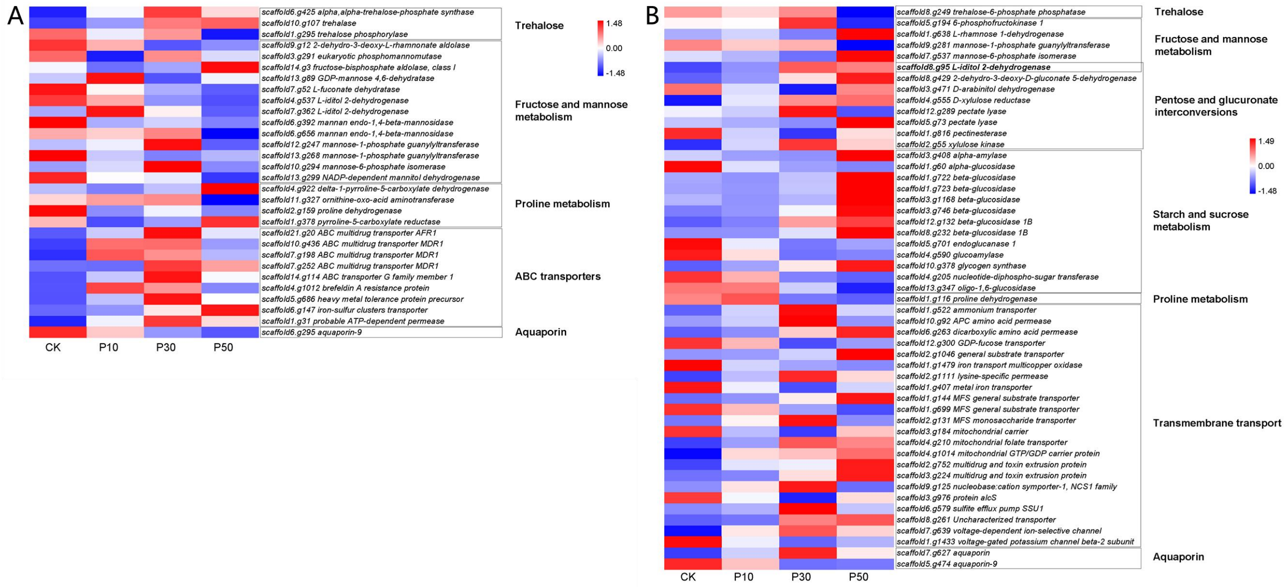

Figure S8. Osmoregulation DEGs that might be related to the drought stress. (A) *Inonotus hispidus*, (B) *Inocutis levis*.

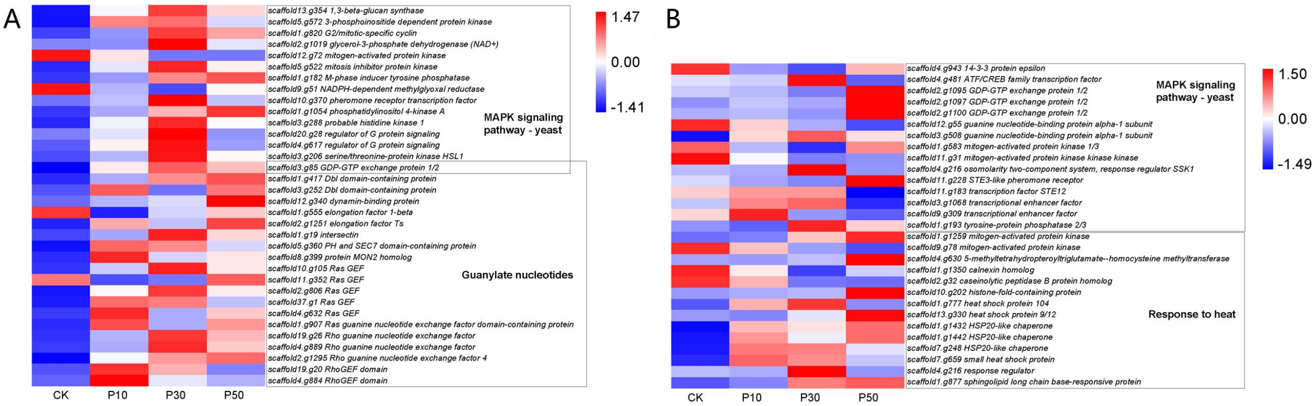

Figure S9. Signal transduction DEGs that might be related to the drought stress. (A) *Inonotus hispidus*, (B) *Inocutis levis*.
